# WINPEPI updated: computer programs for epidemiologists, and their teaching potential

**DOI:** 10.1186/1742-5573-8-1

**Published:** 2011-02-02

**Authors:** Joseph H Abramson

**Affiliations:** 1 School of Public Health and Community Medicine, Hebrew University, Jerusalem, Israel

## Abstract

**Background:**

The WINPEPI computer programs for epidemiologists are designed for use in practice and research in the health field and as learning or teaching aids. The programs are free, and can be downloaded from the Internet. Numerous additions have been made in recent years.

**Implementation:**

There are now seven WINPEPI programs: DESCRIBE, for use in descriptive epidemiology; COMPARE2, for use in comparisons of two independent groups or samples; PAIRSetc, for use in comparisons of paired and other matched observations; LOGISTIC, for logistic regression analysis; POISSON, for Poisson regression analysis; WHATIS, a "ready reckoner" utility program; and ETCETERA, for miscellaneous other procedures. The programs now contain 122 modules, each of which provides a number, sometimes a large number, of statistical procedures. The programs are accompanied by a Finder that indicates which modules are appropriate for different purposes. The manuals explain the uses, limitations and applicability of the procedures, and furnish formulae and references.

**Conclusions:**

WINPEPI is a handy resource for a wide variety of statistical routines used by epidemiologists. Because of its ready availability, portability, ease of use, and versatility, WINPEPI has a considerable potential as a learning and teaching aid, both with respect to practical procedures in the planning and analysis of epidemiological studies, and with respect to important epidemiological concepts. It can also be used as an aid in the teaching of general basic statistics.

## Introduction

Numerous procedures have been added to the WINPEPI freeware computer programs for epidemiologists since the publication of a description of the package in 2004 [1]. The number of programs (now all 32-bit) has grown from four to seven, and the total number of modules (most of which provide multiple procedures) from 75 to 122. This paper describes the current version (11.0) of the package, with emphasis on recent enhancements and the package's potential as a learning or teaching aid.

WINPEPI is the Windows version of the DOS-based PEPI (an acronym for *P*rograms for *EPI*demiologists) package [2], which grew from a set of programs for programmable pocket calculators published in 1983 [3]. It is not a complete compendium of statistical routines for epidemiologists. It does not, for example, provide Cox regression, procedures for the study of disease clustering, or propensity-score methods. Nor does it provide data-management facilities. But it is a handy resource for many commonly-used routines and for others that are not very commonly used or not very easily found (or not easily found in a user-friendly format). Reviewers have repeatedly called it a "Swiss army knife" of utilities for epidemiologists and biomedical researchers.

## The programs

The programs are DESCRIBE, COMPARE2, PAIRSetc, WHATIS, and the recently-added LOGISTIC, POISSON, and ETCETERA. They are accompanied by detailed manuals (occupying over 500 pages in all), which explain the uses and limitations of specific procedures, and furnish formulae and references.

### Describe

DESCRIBE now contains 21 modules for use in descriptive epidemiology, including the appraisal of separate samples in comparative studies. It can appraise rates or proportions or categorical data (dichotomous, nominal or ordinal) or numerical data (including survival data), examine a sequence of rates or other values (including the appraisal of seasonal variation), perform direct and indirect standardization, estimate prevalence from a cluster or stratified sample or from pooled samples or by the capture-recapture method, determine required sample sizes, and appraise screening or diagnostic tests (including meta-analytic procedures).

Recent enhancements include plotting of epidemic curves using data or a model; probit analysis and a nonparametric counterpart; and improved procedures concerning diagnostic and screening tests - appraisals, comparisons, combination, meta-analyses, chance-corrected measures of validity, stratum-specific likelihood ratios, determination of optimal cutpoints, and determination of required sample size.

### Compare2

COMPARE2 (now with 30 modules) is for use in comparisons of two independent groups or samples. It can handle both categorical data (dichotomous, nominal or ordinal; including clustered binomial data) and numerical data (including survival times), proportions or odds, risks, and rates, and determine power and sample size for a variety of tests. It can deal with stratified data, permitting the control of confounding by the stratifying variables, and the assessment of heterogeneity indicative of effect modification. It may be used for analyses and meta-analyses of cross-sectional, cohort and case-control studies and trials.

Recent enhancements include the drawing of forest plots, and two tests for a skewed funnel plot (for use in meta-analysis); added or modified procedures for comparing trimmed means, for comparing ordered categories, for appraising dose-response relationships and other trends, for comparing variances, for comparing the changes in two groups, for appraising equivalence, for appraising differences in one tail of a distribution, and for analyzing data based on inverse sampling; ridit analysis of a 2 × *k *table with ordered categories; pairwise comparisons of categories in a 2 by *k *table; additional measures of effect (*omega*-squared, *eta*-squared, Cohen's *d *and *w*, and standardized mean differences and ratios of means); confidence intervals for the generalized odds ratio for ordered categories; pooled results for stratified data in survival analyses, and directly-standardized survival proportions to control for confounding; sample sizes for cluster-randomization trials; and the calculation of required sample size from the results of a previous comparison.

### Pairsetc

PAIRSetc (now with 34 modules) performs comparisons of paired and other matched observations, such as matched-control trials and cohort studies, matched case-control studies, and before-after studies, and reliability studies that compare two or more replicate observations or methods of measurement. The "etc" in its name indicates its ability to deal with matched sets larger than pairs. It may be used for analyses and meta-analyses of cross-sectional, cohort or case-control studies, and trials. Like COMPARE2, it can handle dichotomous, nominal, ordinal, or numerical variables (including survival times), and can analyze stratified data. It can appraise the effect of misclassification, and determine power and sample size for a variety of tests.

The many recent enhancements include two-way analysis of variance by ranks, analysis of covariance, monotonic regression analysis, the analysis of paired survival data, assessment of regression to the mean, tests that adjust for this regression, measures of effect (*omega*-squared, Cohen's *f*, generalized odds ratio for ordered categories), analysis of clustered matched-pair data, analysis of marginal homogeneity, procedures for using incompletely paired data when comparing proportions, analysis of data obtained by inverse sampling, and the determination of sample sizes for estimating *kappa *and for McNemar tests. For analyses of agreement, there are added or modified procedures with respect to the analysis of variable as well as fixed numbers of replicate measurements; weighted *kappa *for multiple ratings; the concurrent appraisal of interrater and intrarater reliability; intraclass correlation coefficients; coefficients of individual agreement; indices of proportionate positive and negative agreement; confidence intervals for percentages of positive and negative agreement; chance-corrected (*delta*-based) measures of agreement; Bayesian confidence intervals for indices of positive and negative agreement; 95% limits of agreement between methods of measurement; tetrachoric correlation coefficients; correlation when some data are misssing; pairwise comparisons of multiple sets of data; and comparisons of intrarater reliability coefficients. There is also a measure of disagreement between numerical observations.

### Whatis

WHATIS is a "ready reckoner" utility program with four modules. It provides a calculator (expression evaluator) that can store constants, interim results, and formulae, enabling them to be recalled when needed; it computes confidence intervals for a variety of statistics; it provides P values corresponding to given values of *z, t, chi*-square, and *F*, and vice versa; and it calculates time lapses between two dates.

The program can now also calibrate a P value so as to provide the Bayesian minimum posterior probability of the null hypothesis.

### Logistic

LOGISTIC performs unconditional or conditional multiple logistic regression analysis, based on simple or categorical variables, using individual or grouped data; the logistic model can include first-degree interactions. A number of indicators of the aptness of the logistic model are provided, including a goodness-of-fit test, the coefficient of discrimination, and other indicators of the discriminatory power of the model. The program can use the findings to estimate the probability of the outcome, odds ratios comparing different sets of values, risk ratios, risk differences, and the number needed to treat.

### Poisson

POISSON performs multiple Poisson regression analysis. It provides various indicators of goodness of fit with the Poisson model. including the deviance and a graphic display of the relationship between standardized residuals and their expected normal scores; and it can use the regression coefficients to compute a rate ratio expressing the contrast between two selected sets of values

### Etcetera

As its name indicates, the new ETCETERA program (which has 31 modules) offers a hotchpotch of procedures. It provides random numbers, various random sampling and randomization (simple, stratified or balanced) procedures, and an aid to random allocation by minimization. It supplies five procedures for adjusting P values derived from multiple significance tests. It can appraise the internal consistency and discriminatory power of a scale based on the sum of scores allotted to its constituent items, computing Cronbach's *alpha *and other coefficients, and indices of conformity with a Guttman scale. It can appraise statistical synergism and antagonism, providing tests for interaction and several indices of synergism. It can be used for comparisons of three or more samples and for the analysis of large contingency tables, supplying a number of measures of association and significance tests, and for the analysis of three-way tables by fitting and testing loglinear models. It can analyse factorial designs and crossover studies. It can apply a median or mean polish procedure to a two-way table, fitting and testing additive or multiplicative effects. It can conduct simple and multiple linear regression, and provides many tools for the analysis of correlation coefficients. It can perform a sensitivity analysis to appraise the effect of hypothetical unmeasured confounders on the strength of an observed association. And it has two modules that use Bayesian methods to aid in the appraisal of associations.

### Operation

**Figure 1:**
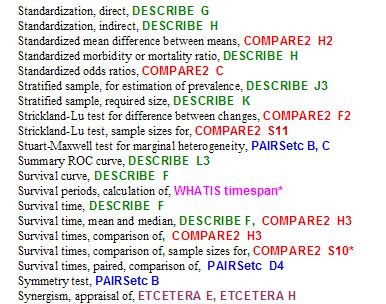
**Excerpt from the PEPI Finder**.

The programs are easy to operate. The installation program puts the programs and manuals in any chosen folder, and places a portal (Figure 1) on the desktop. (In Windows 7, it may be convenient to pin the portal to the Start menu or the Taskbar.) Updating is simple and rapid; the installation program merely overwrites the older programs and manuals.

**Figure 2:**
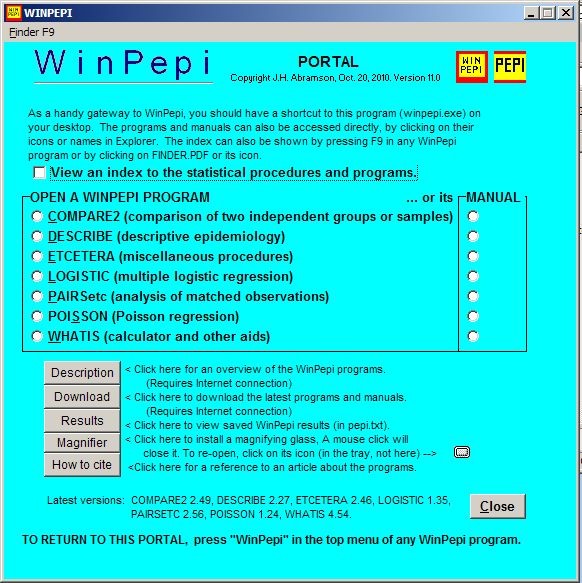
**WNPEPI portal**.

The portal provides access to all the programs and their manuals, and to a Finder. The Finder is an alphabetical index that now has over 700 entries (statistical procedures, measures, and kinds of study), and indicates which programs and modules are appropriate for each purpose; it also serves as an index to the manuals. As may be seen in the excerpt shown in Figure 2, the programs are colour-coded. The Finder may point to more than one module; the entry for " Survival time, mean and median", for example, indicates that module F of DESCRIBE and module H3 of COMPARE2 may both be appropriate. When a required program has been identified (which of course requires the user to know what he or she wants), it can be accessed from the portal.

The programs can also be opened by clicking on their icons; they can be run from a portable device such as a USB flash drive.

**Figure 3:**
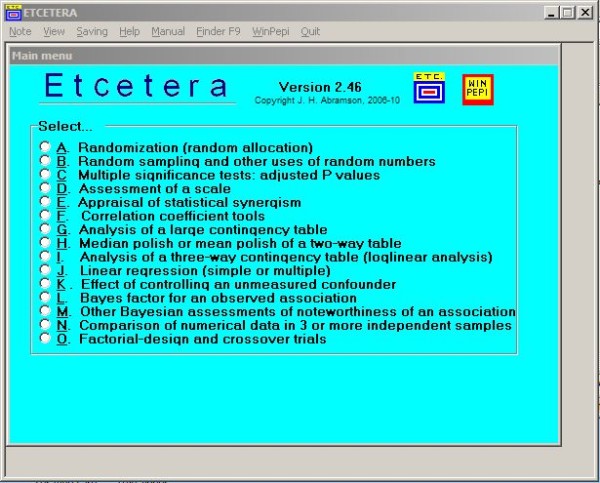
**ETCETERA: Opening screen**.

When a program has been opened, a click selects the appropriate module (see Figure 3), and one or more options may be selected (if offered). A data-entry screen then appears. Two of the new programs can read data files; but in most instances, data must be entered at the keyboard or pasted from a text file or spreadsheet (this usually requires prior counting and summarization, either manually or by using statistical software that processes primary data). In the interest of user-friendliness, alternative forms of data are often accepted, e.g. numerators instead of rates or proportions, and either individual or grouped observations. Also, simple on-screen instructions, pop-up hints, and help screens are provided. If required, the program's manual and the Winpepi portal can now be accessed directly from the program. Warning messages are shown if obvious errors are made when entering data or if essential items are omitted.

The results shown on the screen are automatically sent to the Windows clip-board, and can thus be immediately pasted to a text file. By default, all results are saved in a disk file, which can be accessed from the WINPEPI portal. A "print" option is provided, as are an option for typing in comments to be pasted, printed, or saved with the results, and an option for viewing all the results previously reported in the current session. There is also a "repeat" option, permitting repetition of the analysis with modification of the data or the analysis options, facilitating sensitivity analyses.

Graphic results are now displayed by 12 of the modules; they can be pasted or printed. In most of the graphs, numerical values can be read by mouse-clicking at any location, optionally after magnifying a segment (zooming).

The portal now also offers a magnifier, for the benefit of users with poor vision or small monitors.

It should not take more than a few minutes to learn how to use the Finder, find and open a program module, and enter data. There is no steep learning curve (or indeed a learning curve at all?), as there is for some other software. (Experience with MINITAB, a package designed with education in mind, is that novitiates "mostly have little difficulty after only 3 hours of instruction" [4].)

When the program has been run, the only remaining hurdle is possible confusion by the multiplicity of results. As a matter of policy, the WINPEPI programs generally perform as many as possible of the potentially useful procedures that the data will permit, without making avoidable demands on the user to specify requirements. As a consequence, they generally display many more results than a specific user requires. They often provide alternative tests (both parametric and nonparametric) and alternative measures of effect, often with confidence intervals estimated by alternative methods.

If, for example, a 4 × 5 table is entered in module G of ETCETERA ("Analysis of a large contingency table") in order (say) to obtain a *chi*-square test, the output will include not only Pearson and likelihood-ratio *chi*-square tests, but also adjusted residuals showing which cells contribute most to *chi*-square, pairwise comparisons of columns and of ranks, a test for trend, a Kruskal-Wallis analysis of variance by ranks, and many measures of association (Cramer's *V*, Sakoda's contingency coefficient, Theil's uncertainty coefficient, 20 odds ratios, Spearman's and Kendall's rank correlation coefficients, Goodman and Kruskal's *gamma*, the general odds ratio and the general risk difference), some with 90%, 95%, and 99% confidence intervals. Or if five sets of numbers are entered in module N1 of ETCETERA ("Comparison of numerical data in 3 or more independent samples"), the output includes the five means and standard deviations, alternative estimates of 90%, 95%, and 99% confidence intervals for the means, an analysis of variance, a test for the homogeneity of variances, measures of magnitude of effect (*omega*-squared, adjusted *eta*-squared, and Cohen's *f *index), the pairwise differences between means and their 90%. 95%, and 99% confidence intervals, significance tests for the pairwise differences between means and for comparisons with one selected group (including tests that take account of multiple testing), a test for the linear trend of means, a measure of the slope of this trend, and the proportion of variability explained by the trend.

Results that one user may find relevant and useful may be useless to another. As stated in a review of epidemiological software [5], "what one person might call 'statistical clutter' might be desirable to other people or even to that person if the person learned about that statistic". Some users may find WINPEPI's rich output stimulating. But those who find it confusing are advised to scroll down until they find the result they wanted, and ignore everything else. Every WINPEPI manual carries the admonition: "This program offers more options than most users will ever need, and will usually display more results than are needed. Ignore the options and results you don't require".

## Learning and teaching potential

Practical experience in the performance of statistical procedures is a useful element in any statistics course. Some would say it is essential - "To arrange a basic statistics course without some inclusion of computer exercises is just out of the question" [6]. The use of statistical software can relieve students of the computational drudgery usually associated with statistics, and enable them to focus on concepts rather than on arithmetic, algebra, and elaborate formulae. This practical experience is important in epidemiology courses also, where "the use of statistical methods in epidemiology can hardly be taught without the practical drill that systematic exercises provide" [7].

WINPEPI's ready availability, its freedom from cost, its portability, its ease of use, its versatility, and its provision of comparable alternative tests and measures make it a suitable candidate for these purposes.

The variety of results in the program outputs can be an advantage. A user wishing to compute *kappa*, for example, can learn from the output that *kappa *has a ceiling value, or that its value can be adjusted to avoid paradoxical results, or that there are other measures that may be helpful.

The programs' manuals, which describe the procedures and their uses and limitations, may be a helpful resource, although they are not written as textbooks. Their literature references may be helpful.

For medical students and other students in the health professions, the aim is to learn or teach epidemiology and the uses of statistics in epidemiology, rather than to learn or teach statistics. WINPEPI has special potential as an aid for this purpose, since (unlike major general-purpose statistical packages) it uses epidemiological terms, and it concentrates on procedures and results that are meaningful to epidemiologists. In a reviewer's words, "PEPI facilitates a ready understanding of important epidemiologic concepts, unfettered by the complexities of statistical programming" [8].

Every statistics course has its own teaching objectives. But a recent guide to the teaching of statistics in epidemiology [6] lists 27 teaching objectives, over half of which refer to "understanding" things, i.e. concepts, rather than "how to do" things. All the "how to do" items that are listed, which include (for example) the computation of standardized rates, the analysis of stratified data, and the measurement of population attributable risks, can be done by WINPEPI.

When considering teaching objectives, emphasis should be given not to the needs of future epidemiological cutting-edge researchers into causes and effects - a minority group who will generally work in academic settings and have ample opportunity to obtain advanced statistical training and statistical advice and help - but to the students who will become rank-and-file health care professionals in clinical or public health contexts [9]. These may or may not themselves do epidemiologic studies, but they will almost certainly try to use the findings of such studies as a basis for their decisions about the health care of individual patients or of groups or populations. It is a *sine qua non *that they should acquire a capacity for the critical appraisal of studies - an ability to judge their quality, the validity of their findings and the inferences drawn from them, and their generalizability and relevance.

Some of these practitioners can be expected to conduct epidemiologic investigations that will provide a basis for practical decisions concerning health care, such as studies of the health needs of a specific group or population, or studies of the correlates of health service utilization, or of trends in the occurrence of a disease or risk factor, or studies of a specific health program's effectiveness. These will generally be observational studies; to the extent that they are concerned with etiology, they will usually endeavour to assess the impact of known causal factors rather than try to find new ones. The basic requirements are an ability to plan sound studies that are free of avoidable flaws that will impair their internal or external validity, and an ability to use simple descriptive and analytical procedures and make sound inferences from their results.

Students who will have to make decisions about the clinical care of patients may be expected to use an evidence-based approach to issues of diagnosis, treatment, and prognosis. "It is almost impossible to teach most of clinical epidemiology without concepts of probability and statistics" [10].

The important epidemiologic concepts that it is hoped to impart, then, have to do with the validity of findings - that is, with uncertainties due to chance, bias, or confounding - and the validity of inferences drawn from the findings; and the important practical procedures that they should learn have to do mainly with clinical care and with the planning and analysis of observational studies. The learning of concepts and of procedures can of course not be separated. Rather, it may be hoped that performance of the statistical procedures will provide a basis for the exploration, discussion and understanding of concepts.

WINPEPI can provide hands-on experience in numerous procedures for the analysis of descriptive and analytic studies. It also provides a variety of aids to the planning and preparation of studies - the estimation of required sample size and power, the selection of random samples, randomization, and appraisal of the validity of measures and the reliability of measurements.

This emphasis on epidemiology does of course not preclude the use of WINPEPI as an aid in the teaching of general basic statistics. The package provides many effect measures - *omega*-squared and *eta*-squared, for example - that are more commonly used in non-epidemiological than in epidemiological studies. WINPEPI's use of epidemiological terms, such as "cases" and "controls", or "exposed" and "not exposed", will have to be taken into account. But the use of these terms is not necessarily a disadvantage. On the contrary, it has been suggested that epidemiology provides a useful context for teaching statistical principles and methods, both because its relevance to real-life problems motivates students, and because epidemiological analyses illustrate basic statistical principles and the role of statistics in distinguishing between association and causality [11].

Here are some specific examples of ways in which WINPEPI programs could be used as aids in the teaching of epidemiology and the uses of statistics in epidemiology.

• DESCRIBE provides numerous indices of the validity of measures - especially of screening and diagnostic tests and risk markers (both "yes-no" measures and those that provide a range of results) and can show how differences in prevalence affect predictive value. It supplies all the statistical tools commonly used by clinical epidemiologists in patient care.

• ETCETERA's misclassification procedures illustrate the importance of taking account of validity. They show, for example, that if a measure with a sensitivity and specificity of only 90% is used, an observed prevalence of 12% points to a true prevalence of 2.5%, and an observed risk ratio of 1.5 (18% versus 12%) can point to a true risk ratio of 7.

•PAIRSetc can assess the reliability of measures, including the appraisal of interrater and intrarater reliability; and ETCETERA can appraise the internal consistency and discriminatory capacity of clinical and other scales based on the scores for their component items.

• All WINPEPI programs give emphasis to measures of effect and their confidence intervals, and demonstrate that confidence intervals are far more informative than P values or "significant"/"not significant" decisions. The sometimes disparate results that COMPARE2's modules for comparing two samples report for different "exact" tests and "exact" estimates of confidence intervals, and for other tests and confidence intervals, may lead to a healthy scepticism. "Exact" methods are not necessarily better than other methods [12].

• ETCETERA's "multiple significance tests" procedures demonstrate that significance may be spurious if multiple tests are done on the same data.

• The modules (in ETCETERA and WHATIS) that use Bayes factors to appraise whether associations are worthy of note can make students aware that the usual significance tests and other statistical procedures may be misleading, in the opinion of proponents of Bayesian statistics, who urge that the Bayesian perspective should be included in elementary statistics courses [13].

• COMPARE2 can appraise the likelihood that the strength of an association is influenced by hypothetical unmeasured confounders.

• COMPARE2's Mantel-Haenszel stratified-data procedures provide a basis for learning how to assess confounding and effect modification and to control for confounding and how to appraise the usefulness of a summary odds or risk ratio, and an understanding of the different uses of odds ratios, risk or rate ratios, and risk or rate differences. "The core of epidemiologic analytic thinking," says Rothman [14], "can be conveyed in a thorough discussion of stratified analysis". This can provide a useful preliminary to the understanding of logistic regression findings. (It may be instructive to feed the same data into COMPARE2 and, afterwards, LOGISTIC.)

• The Hosmer-Lemeshow goodness-of-fit test provided by LOGISTIC draws attention to the possibility that a logistic model is not necessarily an appropriate one. This may engender scepticism about published inferences from logistic regression analyses that omit this test.

• ETCETERA demonstrates that random allocation produces groups that are not necessarily equivalent, but have random differences that may lead to confounding effects, by reporting the proportions of odd-numbered subjects in groups selected by simple randomization.

• COMPARE2 has a module that shows how regression to the mean can produce spurious evidence of change in a "before-after" study.

• DESCRIBE can draw a survival curve that shows the maximal possible bias (in either direction) that may be caused by dropouts.

• COMPARE2 can take account of missing values in an analysis of a 2 × 2 table, and PAIRSetc can compare paired observations when some pairs are incomplete. Depending on the reasons for "missingness", the inclusion of incomplete pairs may reduce bias [15].

• DESCRIBE offers world, European and African standard populations for use in age standardization, and can also use age intervals as weights, dispensing with the need for a standard population [16].

• DESCRIBE can estimate prevalence and its confidence intervals from cluster samples, stratified samples, and pooled samples.

• DESCRIBE can use a capture-recapture method to estimate the total number of cases from overlapping incomplete lists, and estimate the exhaustiveness of each list.

• COMPARE2's meta-analytic procedures appraise the possible effect of publication bias, and conduct sensitivity analyses to detect studies that may have an undue influence on the findings.

## Discussion

WINPEPI's advantages are its ready availability (free of cost), its user-friendliness, and its rich content. Apart from its utilitarian value (increased by its recent enhancements), it can be used as a teaching aid. At least two textbooks currently make extensive use of WINPEPI [17,18].

Apart from the possibly confusing multiplicity of procedures (a problem that the Finder should minimize) and of results, WINPEPI's main limitations are the absence of data-management facilities, and the need to enter data at the keyboard or by pasting from a text file or a spreadsheet.

There are many software packages that can prepare the data for analysis, including the freeware packages EpiData [19] and Epi Info [20].

Data entry at the keyboard or by pasting can sometimes be an advantage, rather than a liability. In the words of a reviewer, "conventional statistical software packages ... are not always helpful when you need to ... rapidly perform simple analyses. For instance, you may want to replicate some analyses from a journal article and compute a Mantel-Haenszel odds ratio, or you may want to compute the sample size for your study while writing a grant proposal. Maybe you want to demonstrate to your students the impact of increasing sample size on the confidence intervals of a proportion. Perhaps you are a student and would like to do your epidemiology or biostatistics homework with some easy-to-use analytical routines... It is in this niche area that PEPI scores!" [8].

For epidemiologists whose data are prepared and stored by, say, one of the popular commercial software packages, and who are well versed in the use of that package, WINPEPI may be seen as a complementary package, for use when it offers analyses not provided by the other package, or when simple rapid analyses not requiring a data set are required, or for teaching purposes.

## Availability and requirements

The latest version of the WINPEPI package (including the programs, their manuals, and the Finder) is always available at http://www.brixtonhealth.com for free download.

The programs and manuals are copyrighted, but may be freely copied and distributed for personal use; they may not be exploited commercially without permission.

The programs are written in Delphi version 5 (32-bit), and can be run in any version of Windows except Windows 3. The manuals are in PDF format, and can be read or printed with (for example) Adobe Acrobat or Foxit Reader.

## Competing interests

The author wrote the WINPEPI programs and manuals, and may be biased in their favour.
